# First complete-genome documentation of HIV-1 intersubtype superinfection with transmissions of diverse recombinants over time to five recipients

**DOI:** 10.1371/journal.ppat.1009258

**Published:** 2021-02-12

**Authors:** Yang Gao, Shan He, Wen Tian, Dan Li, Minghui An, Bin Zhao, Haibo Ding, Junjie Xu, Zhenxing Chu, Hong Shang, Xiaoxu Han

**Affiliations:** 1 NHC Key Laboratory of AIDS Immunology (China Medical University), National Clinical Research Center for Laboratory Medicine, The First Affiliated Hospital of China Medical University, Shenyang, China; 2 Key Laboratory of AIDS Immunology, Chinese Academy of Medical Sciences, Shenyang, China; 3 Key Laboratory of AIDS Immunology of Liaoning Province, Shenyang, China; 4 Collaborative Innovation Center for Diagnosis and Treatment of Infectious Diseases, Hangzhou, China; 5 Jilin Cancer Hospital, Changchun, China; University of North Carolina at Chapel Hill, UNITED STATES

## Abstract

Human immunodeficiency virus type 1 (HIV-1) recombinants in the world are believed to be generated through recombination between distinct HIV-1 strains among coinfection or superinfection cases. However, direct evidence to support transmission of HIV-1 recombinants from a coinfected/superinfected donor to putative recipient is lacking. Here, we report on the origin and evolutionary relationship between a set of recombinants from a CRF01_AE/CRF07_BC superinfected putative donor and diverse CRF01_AE/CRF07_BC recombinants from five putative recipients. Interviews on sociodemographic characteristics and sexual behaviors for these six HIV-1-infected men who have sex with men showed that they had similar ways of partner seeking: online dating sites and social circles. Phylogenetic and recombination analyses demonstrated that the near-full-length genome sequences from six patients formed a monophyletic cluster different from known HIV-1 genotypes in maximum likelihood phylogenetic trees, were all composed of CRF01_AE and CRF07_BC fragments with two common breakpoints on *env*, and shared 4–7 breakpoints with each other. Moreover, 3’ half-genomes of recombinant strains from five recipients had identical/similar recombinant structures with strains at longitudinal samples from the superinfected donor. Recombinants from the donor were paraphyletic, whereas five recipients were monophyletic or polyphyletic in the maximum clade credibility tree. Bayesian analyses confirmed that the estimated time to the most recent common ancestor (tMRCA) of CRF01_AE and CRF07_BC strains of the donor was 2009.2 and 2010.7, respectively, and all were earlier than the emergence of recombinants from five recipients. Our results demonstrated that the closely related unique recombinant forms of HIV-1 might be the descendent of a series of recombinants generated gradually in a superinfected patient. This finding highlights the importance of early initiation of antiretroviral therapy as well as tracing and testing of partners in patients with multiple HIV-1 infection.

## Author summary

Recombination is a major mechanism for rapid evolution and diversification of human immunodeficiency virus type 1 (HIV-1). Multiple HIV-1 infection is believed to be a prerequisite for generating new recombinant strains. Recombinants sharing some breakpoints might have a common ancestor or direct parental/progenitor relationship or be irrelevant in evolution. However, evidence to support transmission of HIV-1 recombinants from coinfected/superinfected donor to putative recipients with recombinant descendants is lacking. Here, we report on a set of HIV-1 CRF01_AE/CRF07_BC unique recombinant forms (URFs) from six HIV-1-infected men who have sex with men (MSM) in China with similar recombination structures and homologous parental strains. Moreover, the 3’ half-genomes of all recombinants from five recipients had similar or identical forms with earlier continuous samples of a superinfected donor, and presented paraphyletic-monophyletic or paraphyletic-polyphyletic relationships in maximum clade credibility trees. These data suggest that HIV-1 superinfection might be a potential source of transmission of a series of closely related HIV-1 CRF01_AE/CRF07_BC URFs. Our study emphasizes the importance of continuous surveillance and early initiation of antiretroviral therapy in HIV-1-superinfected individuals with active high-risk behavior and substance abuse to prevent the generation and spread of recombinant strains among HIV-infected individuals.

## Introduction

Human immunodeficiency virus type 1 (HIV-1) is characterized by extensive genetic diversity. Recombination is a major mechanism for the rapid evolution and diversification of HIV-1 [1]. More than 100 circulating recombinant forms (CRFs), along with massive unique recombinant forms (URFs) in HIV-1 group M have been identified worldwide [2] (www.hiv.lanl.gov). Some recombinants have recombined further with other subtypes or CRFs to generate second-generation recombinants [3]. It has been estimated that HIV-1 recombinants, including CRFs (16.7%) and URFs (6.1%), accounted for 22.8% of epidemics globally between 2010 and 2015 [4]. Recombination of HIV-1 can potentially change biological characteristics, fitness, susceptibility to antiretroviral drugs, disease progression, as well as the diagnostic accuracy of serology- and molecular- based assays [5–8].

Identification of HIV-1 recombinants from primary infected individuals usually indicates the spreading of these recombinants among a population, but the origin and transmission history are incompletely understood [9]. Deciphering the origin of a group of recombinants with high genetic similarity using phylogenetic analyses is difficult. Studies have reported that recombinants sharing some breakpoints might have a common recombinant ancestor (e.g., CRF07_BC and CRF08_BC in China [10,11] and HIV-1 BF intersubtype recombinant viruses in Argentina [12]) or that there might be a direct parental/progenitor relationship between them (e.g., CRF48_01B and CRF74_01B were probably descended from CRF33_01B in Malaysia [13,14]) or that they may be irrelevant in evolution. Common breakpoints may be attributable to fragile sites or hairpin structure of genomic RNA, pause sites, high-pairing probability, or sequence similarity during reverse transcription. In this instance, the breakpoints often occur in well-conserved regions of viral genomes. These potential mechanisms of recombination have been supported by *in vitro* experiments and mathematical models, but they may be more complex *in vivo* [15–18]. Therefore, elucidation of the recombination mechanisms of HIV-1 may help to guide the surveillance and prevention of HIV spread.

Multiple HIV-1 strains infecting the same person concurrently (“coinfection”) or one after another (“superinfection”) is believed to be the prerequisite for the generation of recombinant strains [19–21], which is supported indirectly by the overlap of “hot areas” for HIV recombinants and multiple infections [22–24]. Moreover, recombinants composed of more than two parental viruses have also been found in HIV-1 superinfected cases [25,26]. However, there is no direct evidence to support the transmission of HIV-1 recombinants from coinfection/superinfection cases to a putative recipient with recombinant descendants, let alone the origin and transmission history of a group of HIV recombinant strains with genetic similarity.

We depicted the origin and evolutionary relationship among a group of closely related CRF01_AE/CRF07_BC URFs (0107 URFs) between an HIV-1 intersubtype superinfected donor and five putative CRF01_AE/CRF07_BC-infected recipients. This finding emphasized the importance of early initiation of antiretroviral therapy (ART) as well as tracing and testing of partners with multiple HIV-1 infection to prevent the spread of recombinant strains.

## Results

### Sociodemographic characteristics and sexual behaviors of six HIV-1-infected men who have sex with men (MSM)

Previously, we identified six patients infected with HIV-1 CRF01_AE/CRF07_BC strains in a newly diagnosed cohort of MSM in Liaoning, northeast China. Among them, the donor was diagnosed with recent HIV-1 infection on 3 March 2010 and started ART on 7 January 2014. His viral load was well-controlled to <100 copies/mL after that. He is a high-earning businessman and self-reported as seeking younger male partners via online dating sites and social circles. He reported sexual behaviors with one regular partner and ≥10 casual partners in the past 3 months before the diagnosis of HIV infection. Insertive and receptive positions were adopted when he had sex with other males without a condom. Moreover, methamphetamine and “rush poppers” were used constantly during sex.

All five recipients were diagnosed with HIV infection between 2013 and 2014. Among them (hereafter termed recipient), recipient 1, 2, 3, and 4 had been diagnosed with a recent HIV infection according to the results of limiting antigen (LAg)-avidity enzyme immunoassay (EIA). Recipient 5 was estimated to have become infected with HIV before the end of 2013. Five recipients were all ~10 years younger and had a lower income than that of the donor. They usually sought male partners via online dating sites and social circles. They self-reported to have had sex with 3–15 male partners within the last 3 months before the diagnosis of HIV infection except recipient 3, a “rent boy,” who had 80 commercial partners and one regular partner (recipient 5). When they had sex with other males, recipient 2, 3, and 4 adopted insertive and receptive positions; recipient 1 and 5 preferred insertive and receptive positions, respectively. Recipient 2 and 4 engaged in condom-less anal sex. Besides, recipient 1, 2, and 3 had a history of substance abuse. The sociodemographic characteristics and sexual behaviors of six HIV-1-infected MSM are outlined in Table 1.

**Table 1:** Sociodemographic characteristics, sexual behaviors, and clinical records of six patients diagnosed with HIV infection.

	Donor (LNA819)	Recipient 1 (LN320639)	Recipient 2 (LN320392)	Recipient 3 (LN328575)	Recipient 4 (LN301538)	Recipient 5 (LN328576)
**Year of birth**	1978	1987	1988	1989	1992	1989
**Native place**	Shenyang, LiaoNing	Shenyang, LiaoNing	Shenyang, LiaoNing	Qigihar, HeilongJiang	Shenyang, LiaoNing	DaLian, LiaoNing
**Residence**	Shenyang, LiaoNing	Shenyang, LiaoNing	Shenyang, LiaoNing	Shenyang, LiaoNing	Shenyang, LiaoNing	Shenyang, LiaoNing
**Occupation**	businessmen	staff	freelancer	rent boy	student	staff
**Monthly income (RMB)**	>20,000	3000	unstable income	unstable income	0	6000
**Sexual behaviors in the last three months before HIV infection**						
Methods to seek male sexual partners	Internet friends’ referrals	Internet, social media, friends’ referrals	Internet, social media	Internet, friends’ referrals	Internet	Internet, friends’ referrals
Characteristics of male sexual partners	young	peer	successful male partner and could give him guidance in career	whoremasters	peer have a job and degree	peer
Number and types of male sexual partners	1 regular partner 10 casual partners	3 casual partners	15 casual partners	1 regular partner (Recipient 5) 80 commercial sex partners	2 regular partners 5 casual partners	6 regular partners (one is Recipient 3)
Sexual role	both receptive and insertive	insertive	both receptive and insertive, predominantly insertive	both receptive and insertive	both receptive and insertive	receptive
**Condom use**						
Anal intercourse	seldom	often	seldom	often	seldom	often
Oral intercourse	no	no	no	no	no	no
**Substance abuse**	methamphetamine rush poppers	rush poppers	methamphetamine rush poppers	rush poppers	no	no
**Syphilis**	+	-	+	-	+	-
**LAg-avidity EIA** ^**a**^	Recent	Recent	Recent	Recent	Recent	LT
**Diagnose of HIV infection**	3-Mar-10	1-Apr-13	22-Feb-13	12-May-14	20-Mar-14	12-May-14
**CD4 counts (cells/ul)**	395	284	366	NA^b^	392	NA
**Viral load (copies/ml)**	109333	74500	112000	NA	61500	NA
**Sampling date**^c^	3-Mar-10 to 23-Sep-13	1-Apr-13	28-Feb-13	12-May-14	20-Mar-14	12-May-14
**Start ART**	7-Jan-14	12-Apr-13	7-Apr-13	NA	20-Mar-14	NA

^a^LAg-Avidity EIA, limiting-antigen avidity enzyme immunoassay, the testing results were used to determine the patients were recent infection (<180 days after seroconversions) or long-term infection (LT, >180 days after seroconversions)

^b^NA, data were not available because of lost to follow-up

^c^Sampling date, all the samples were collected from plasmas. Eight plasma samples were collected from donor from 3 March 2010 to 23 September 2013. Five recipients collected only one plasma sample at baseline/seroconversion. The CD4 counts and Viral load of donor were obtained at 29 November 2011.

### Identification of a lineage of HIV-1 CRF01_AE/CRF07_BC URFs among MSM in Liaoning

**Fig 1:**
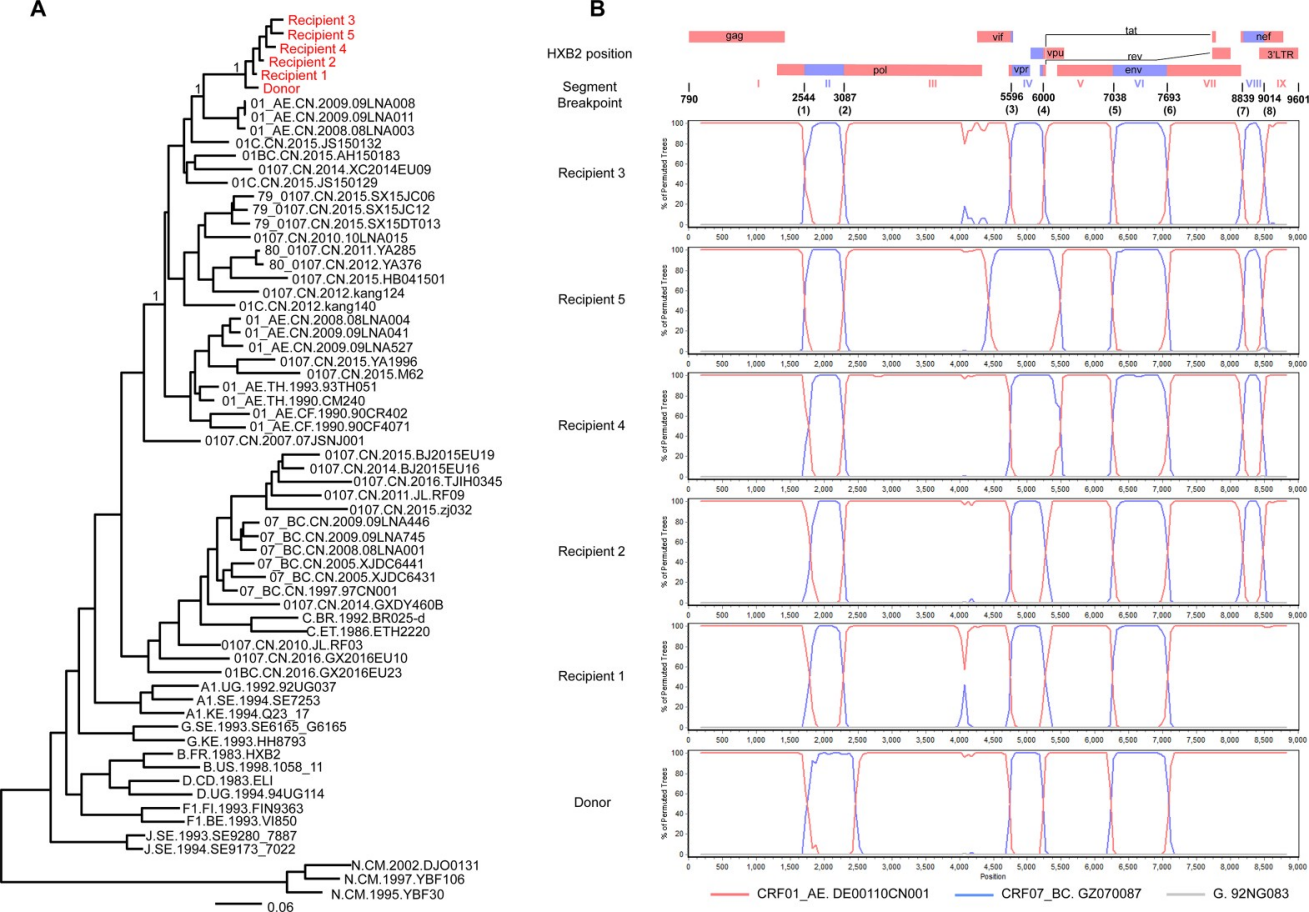
The phylogenetic tree and recombination analysis of NFLG of six HIV-1-infected MSM from Liaoning, northeast China. (A) The phylogenetic analysis of NFLG from six HIV-1-infected MSM (marked red) and reference sequences which were downloaded from HIV database (marked black). Maximum likelihood phylogenetic trees were constructed by Fast Tree and edited by Fig tree v1.4.2. The scale length represents 6% nucleotide sequence divergence. (B) Recombination analysis of six HIV-1 NFLG by using SimPlot (v3.5.1). CRF01_AE and CRF07_BC were used as putative parental sequences according to the online tool HIV Blast and subtype G (14003718) was used as an outgroup. The parameters of analysis were the default except the window size was 350 bp and step size was 50 bp, bootstrap replicate was 250. NFLG, near full-length genomes; MSM, men who have sex with men.

To validate the lineage of HIV-1 new recombinants, the near-full-length genome (NFLG) (HXB2: 790–9601 bp) was used for phylogenetic analyses (Fig 1A). The NFLG of six patients formed a distinct monophyletic cluster that was separate from any other known subtypes, CRFs and URFs in the maximum likelihood (ML) phylogenetic tree with poster probability value 1, suggesting that this was a lineage of new HIV-1 URFs.

### Six HIV-1 CRF01_AE/CRF07_BC URFs showed similar recombination forms and homologous parental strains

The recombination forms along the whole genomes of six strains were first screened with Recombinant Identification Program (RIP) and jumping profile hidden Markov model (jpHMM), and then validated with Simplot Six strains had similar (but not identical) recombination forms (Fig 1B). First, six strains were all CRF01_AE/ CRF07_BC recombinants with a CRF01_AE backbone and three or four CRF07_BC insertions in *pol*, *vpr*, *tat/rev*, *env*, and *nef*. Second, six strains shared two identical breakpoints: the fifth and sixth breakpoint in *env*; five strains shared the first and second breakpoints in *pol* except in the donor; four strains shared the seventh and eighth breakpoint in *nef* except in recipient 1 and the donor. Recipient 1, 2, and 3 and the donor shared the third and fourth breakpoint *in vpr and first exon of tat/rev*. Third, the main difference among recipient 3, 4, and 5 was the length of the CRF07_BC segment IV, which was 404 bp, 970 bp, and 644 bp, respectively.

**Fig 2:**
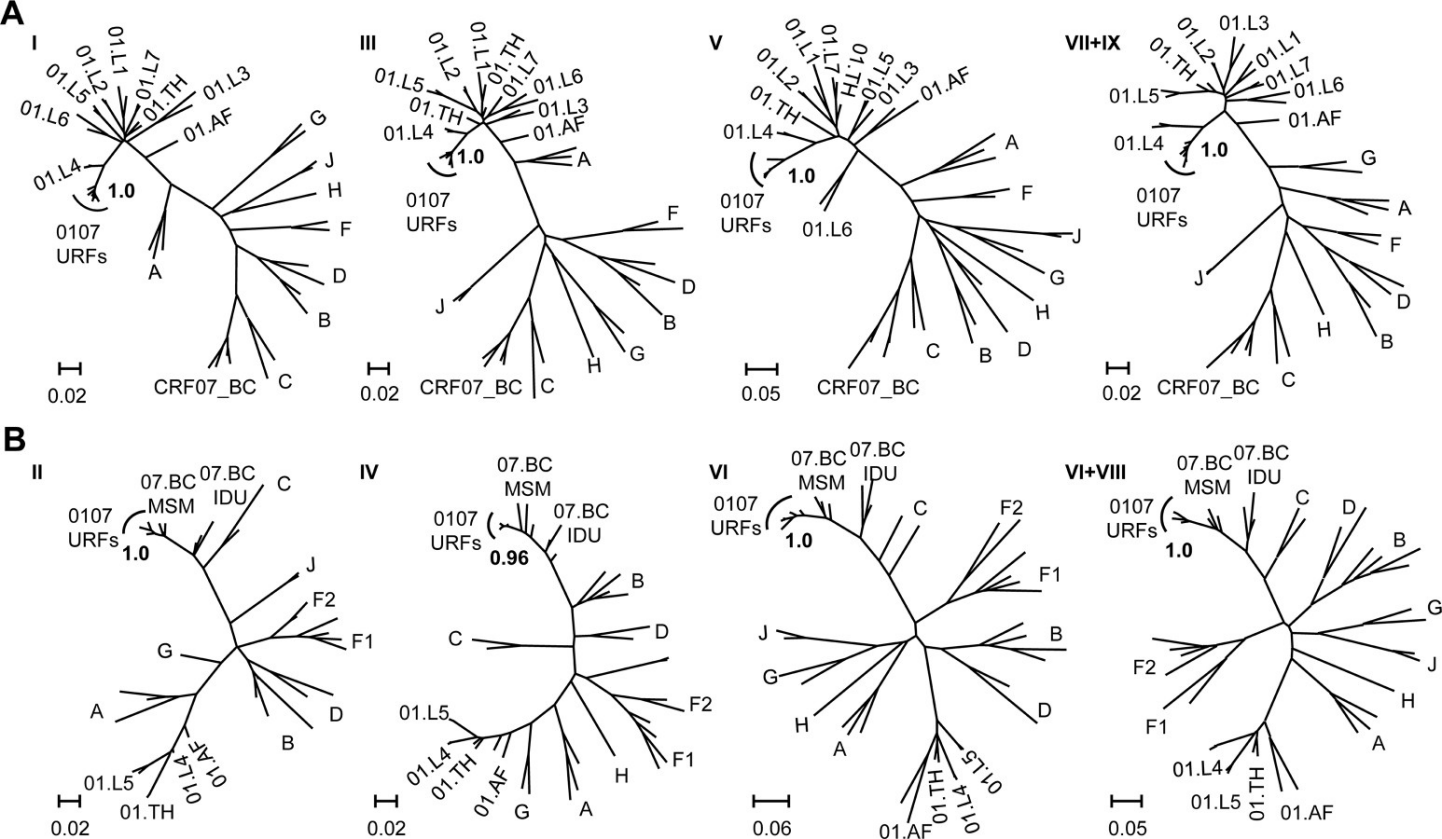
Sub-region phylogenetic analysis of NFLG of six 0107 URFs. Six HIV-1 CRF01_AE/CRF07_BC recombinants were divided into nine segments (I-IX) according to the recombinant breakpoints shown in the HIV-1 structure diagram in Fig 1B. ML trees were constructed by Fast tree and edited by Fig tree v1.4.2, poster probability values over 0.90 were shown at each node. (A) The subtypes of segment I, III, V, VII, IX were CRF01_AE. (B) The subtypes of segment II, IV, VI, VIII were CRF07_BC. The segment VIII and IX were too short in some patients and were combined the segment VI and VII, respectively to construct ML trees. The scale bars were shown at the bottom of each sub-region tree. ML tree, Maximum likelihood phylogenetic trees.

To determine the evolutionary relationship and potential parental strains of the six strains, we undertook phylogenetic analyses on sub-regions between breakpoints (Fig 2). In general, six strains displayed monophyletic clustering in all the sub-region trees of CRF01_AE and CRF07_BC segments. In trees I, III, V, and VII+IX, the CRF01_AE segments from six strains belonged to CRF01_AE lineage 4 among the Chinese MSM population (poster probability = 1) (Fig 2A). In trees II, IV, VI, and VI+VIII, the CRF07_BC segments of six strains belonged to the CRF07_BC lineage predominant among the Chinese MSM population (poster probability ≥0.96) (Fig 2B). Taken together, this high genetic similarity and homologous parental strains suggested a close evolutionary relationship among the six CRF01_AE/CRF07_BC recombinant strains.

### Most likely origin of the series of HIV-1 CRF01_AE/CRF07_BC URFs

**Fig 3:**
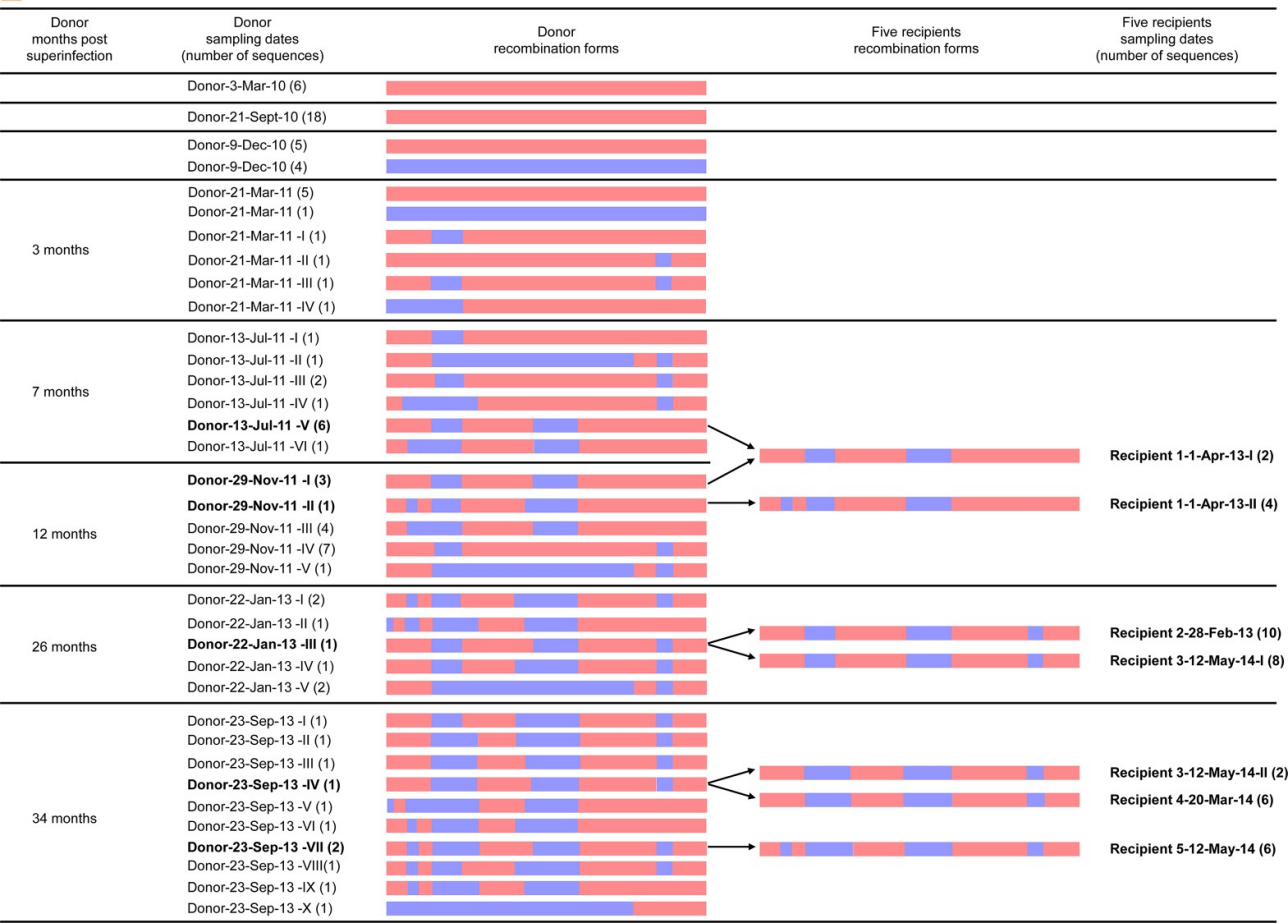
Recombination structure analysis of 3’ half genome sequences (HXB2: 4950-9589nt) from one HIV-1-superinfected donor and five HIV-1 CRF01_AE/CRF07_BC infected recipients. The first column represents the time interval from the first detection of superinfecting virus to this sampling date. The second and fifth columns represent the sampling dates of the donor and five recipients, respectively. The third and fourth columns described the recombination forms of the donor and five recipients, respectively. The sequence name is composed of patient’s ID+ sampling date + recombination forms + (the number of sequences with identical recombination forms at this time point). The different recombination forms at each sampling date are marked by Roman numerals. Initial CRF01_AE strains and superinfecting CRF07_BC strains were marked as light-coral and slate-blue, respectively. The arrow indicates similar or identical recombination structures were identified between the donor and 5 putative recipients.

Among six patients, according to clinical records and results for LAg-avidity EIA, the donor was diagnosed to have been infected with an HIV-1 CRF01_AE strain on 3 March 2010 (Table 1). Moreover, the donor had been identified to have superinfected another CRF07_BC strain in our previous study [27]. To determine the recombination process between the primary infected CRF01_AE strain and superinfected CRF07_BC strain in the donor, the 3′ half-genome was obtained from longitudinal samples by a single-genome amplification (SGA) strategy and used for recombination analyses (Fig 3). The superinfected CRF07_BC strain was obtained first through the SGA strategy detected from the donor on 9 December 2010. The recombination between CRF01_AE and CRF07_BC was first detected ~3 months after superinfection. The predominant CRF01_AE/CRF07_BC recombinants were detected ~7 months and ~12 months after superinfection, respectively. Although the quasispecies and composition of the CRF01_AE/CRF07_BC recombinants among longitudinal samples of the donor changed continuously, some recombinants could be detected at ≥2 time points. Also, some identical/similar breakpoints were detected between distinct recombinants at different time points (S1A and S2 Figs), which suggested continuous evolution of CRF01_AE/CRF07_BC recombinants *in vivo* under immune selection from the host.

We further compared the recombination forms of 3′ half-genomes of the donor at longitudinal samples and five recipients at baseline or after seroconversion (Fig 3). The viral quasispecies of the donor were more complex than those of the five recipients who had only one or two recombination forms. More importantly, each recombinant strain from five patients had at least one similar or identical recombination form with the strain from the donor sample at an earlier time point (Figs 3, and S1B and S2). For example, two forms of recombinants from recipient 1 (1 April 2013) were similar with recombinants from donor samples on 13 July 2011 and 29 November 2011, respectively. Similarly, two forms of recombinants from recipient 3 (12 May 2014) were similar or identical to recombinants from donor samples on 22 January 2013 and 23 September 2013, respectively. Moreover, the recombinants from recipients 2 (28 February 2013), recipient 4 (20 March 2014), and recipient 5 (12 May 2014) all had identical forms with those of earlier longitudinal samples from the donor. These data suggested that recombinants from five recipients were most likely being transmitted from the donor directly or indirectly.

### Temporal evolutionary relationships among HIV-1 CRF01_AE/CRF07_BC URFs

**Fig 4:**
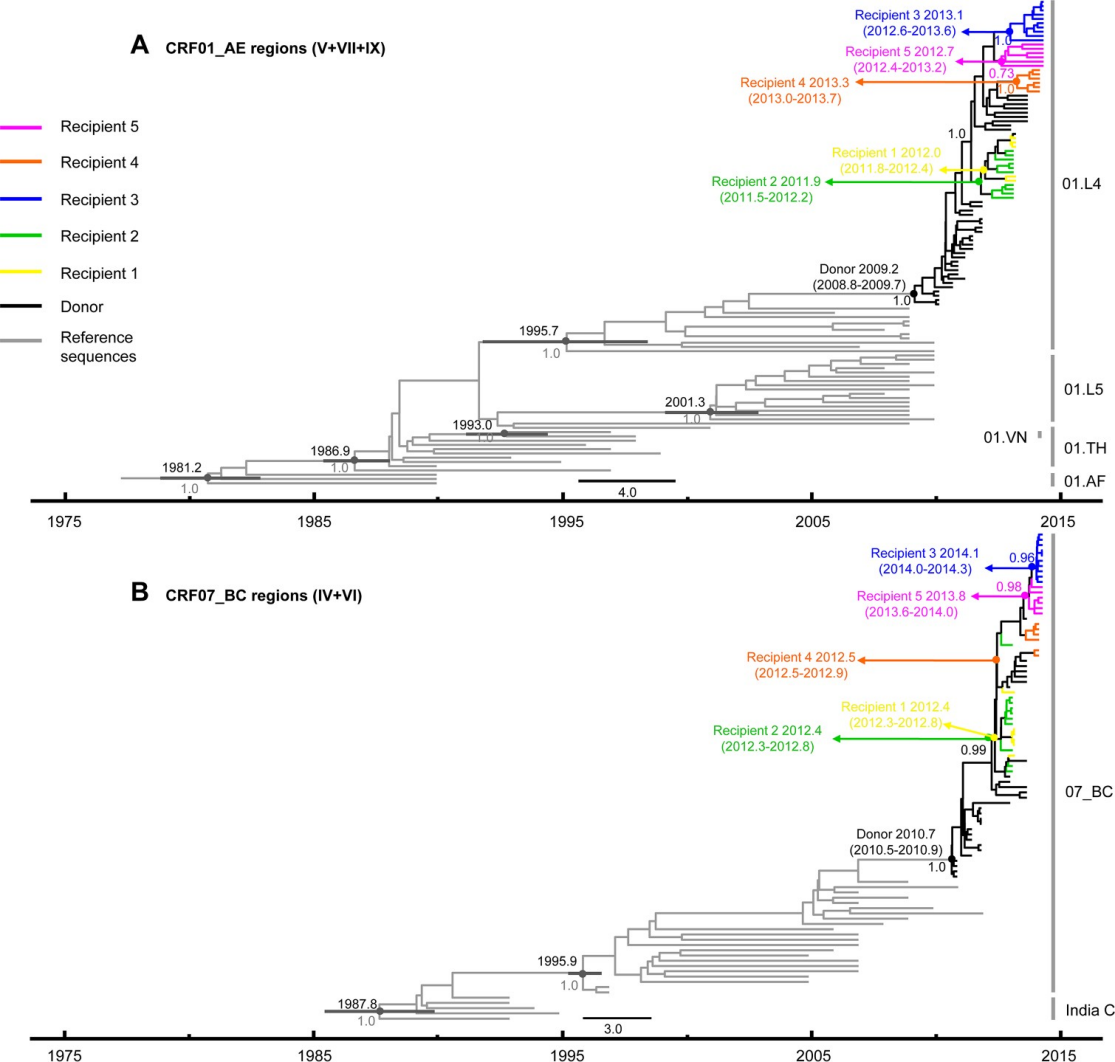
Maximum clade credibility (MCC) trees of HIV-1 CRF01_AE/CRF07_BC recombinants from the donor and five putative recipients. Some representative sequences of the donor, all sequences of five recipients and references were selected to constructed MCC trees. Bayesian molecular clock analysis were performed using BEAST v2.5.1 for concatenated CRF01_AE segments (Regions V+VII + IX) (A) and CRF07_BC segments (Regions IV+VI) (B). The medians of the tMRCA and 95% HPD of nodes relevant to this study were shown as follows: African CRF01_AE (01.AF) ancestor, 1981.2 (1979.3–1983.0); Thai CRF01_AE (01.TH) ancestor, 1986.9 (1985.6–1988.1); Vietnam CRF01_AE (01.VN) ancestor, 1993.0 (1991.6–1994.5); CRF01_AE Lineage 5 (01.L5), 2001.3 (1999.7–2002.7); CRF01_AE Lineage 4 (01.L4), 1995.7 (1993.1–1998.1). C ancestor, 1987.8 (1985.6–1989.8); CRF07_BC ancestor 1995.9 (1995.2–1996.5). The tMRCA of concatenated CRF01_AE segments of the donor at sampling date 13-Jul-2011, 29-Nov-2011, 22-Jan-2013, 23-Sep-2013 are 2010.5 (2010.3–2010.7), 2010.5 (2010.3–2010.7), 2012.1 (2011.8–2012.6), 2011.7 (2011.4–2012.1). The tMRCA of concatenated CRF07_BC segments of the donor at sampling date 13-Jul-2011, 29-Nov-2011, 22-Jan-2013, 23-Sep-2013 are 2011.1 (2010.9–2011.3), 2011.2 (2011.1–2011.5), 2011.2 (2011.0–2011.5), 2012.3 (2012.0–2012.7). tMRCA, time of the most common ancestor; 95% HPD, 95% highest probability density.

To investigate the possible evolutionary relationship between these closely related CRF01_AE/CRF07_BC recombinants from six patients, the concatenated CRF01_AE segments and CRF07_BC segments were analyzed by Bayesian molecular clocks (Fig 4A and 4B). In the maximum clade credibility (MCC) tree of CRF01_AE and CRF07_BC, the sequences from six patients formed a monophyletic cluster within all reference sequences, respectively. Within each cluster, the initial infected CRF01_AE strains and the superinfected CRF07_BC strains from the donor were located at the root. The strains of five recipients formed internal branches following the CRF01_AE strains and CRF07_BC strains from the donor, respectively. Moreover, the strains from the donor were paraphyletic, whereas the strains from the five recipients were monophyletic or polyphyletic in the MCC tree. This result was further supported by the evolutionary ML trees constructed with concatenated CRF01_AE and CRF07_BC segments, respectively (S3 Fig).

The estimated time to the most recent common ancestor (tMRCA) for the concatenated initial infected CRF01_AE strain and superinfected CRF07_BC strain of the donor was dated to 2009.2 (95% highest probability density 2008.8–2009.7) and 2010.7 (2010.5–2010.9), respectively (Fig 4A and 4B). These observations were consistent with the results using LAg-avidity EIA and SGA on estimation and identification of initial infection and superinfection. The tMRCA of the concatenated CRF01_AE segments in the donor (13 July 2011, 29 November 2011, 22 January 2013, and 23 September 2013) was earlier than that of the corresponding similar/identical recombinants of the five recipients. The tMRCA of concatenated CRF01_AE segments of recipient 1, 2, 3, 4, and 5 was estimated to be 2012.0 (2011.8 to 2012.4), 2011.9 (2011.5 to 2012.2), 2013.1 (2012.6 to 2013.6), 2013.3 (2013.0 to 2013.7), and 2012.7 (2012.4 to 2013.2), respectively (Fig 4A). Similar results were found for the tMRCA of CRF07_BC segments of the six patients (Fig 4B).

## Discussion

The rapid increase and high diversity of HIV-1 recombinants are a great challenge for the prevention and surveillance of HIV infection globally. The co-circulation of various strains and high rate of multiple infection are prerequisites for a generation of new HIV-1 recombinants. We identified a lineage of new 0107 URFs with homologous parental strains and similar recombination forms among six HIV-1-infected MSM in northeast China. Epidemiology, quasispecies diversity, timelines, and phylogenetics supported the notion that the potential origin of the ancestral virus of a series of closely related 0107 URFs could be traced back to an HIV-1-superinfected individual.

First, HIV-1-infected MSM carry a higher risk of multiple infection compared with that in heterosexuals [28]. Junjie Xu and colleagues reported that ~35% of MSM had >5 male sexual partners in the last 12 months in Shenyang, Liaoning [29]. In our study, six patients all sought multiple male sexual partners mainly through online dating sites and social circles in the same area. Moreover, most of them seldom used condoms, admitted substance abuse during anal/oral sex with their casual male sexual partners, and had a history of syphilis, which further increased the potential for HIV infection and acquisition of multiple viruses [30–34].

Second, the donor was an HIV-1 CRF01_AE/CRF07_BC-superinfected patient. This patient developed a series of CRF01_AE/CRF07_BC recombinants with 1–5 common breakpoints but non-identical recombination forms in longitudinal samples within a treatment-naïve period of ~4 years. This finding is consistent with the observation by McCutchan and colleagues that various recombinants from a heterosexual superinfected individual in Tanzania had common breakpoints in *gag*, *env*, and *gp41/nef* regions in serial samples within 30 months [26]. Compared with the method used by McCutchan and colleagues to amplify three regions of the HIV (multiple-region hybridization assays), we used the SGA method to amplify the relatively long fragments of the 3’ half-genome, which could fully reflect the complex structure of the recombinants in six patients [35]. Five recipients in our study had one or two genetically homogeneous CRF01_AE/CRF07 recombinants at baseline or after seroconversion, which showed less quasispecies complexity compared with that in the donor. Surprisingly, the recombination forms of all strains from five patients resembled those at different sampling dates from the donor. Studies have demonstrated the loss of genetic diversity of the HIV within donors to new hosts upon sexual transmission [36] and mother-to-child transmission [37], which may result from a “transmission bottleneck”. These data imply that this HIV-1-superinfected patient might have been the donor who transmitted a series of 0107 URFs to new hosts.

Third, according to clinical records and the results of LAg-avidity EIA, we found that the putative donor was diagnosed with HIV infection about 3–4 years earlier than the other five patients, and was the first to be detected with CRF01_AE/CRF07_BC recombinants. The tMRCA of the recombinants of the putative donor was also earlier than that of the other five patients. Furthermore, the putative donor did not start ART until 2014 (i.e., ~4 years after the diagnosis).

Finally, based on Bayesian analyses, the topology of the MCC tree provided compelling evidence of the source of these 0107 URFs. Strains from the putative donor and five recipients formed paraphyletic–polyphyletic or paraphyletic–monophyletic donor-recipient joint phylogeny. Studies have reported that phylogenetic methods might be used to infer the transmission history of epidemiologically linked hosts in an HIV-mono-infected population [38]. Paraphyletic–polyphyletic trees support direct transmission and are believed to exclude intervening transmission and a common source. Typically, paraphyletic–monophyletic trees result from direct or indirect transmission [39].

In recent years, several HIV-1 CRF01_AE and B-related second-generation recombinants have been identified in Asia [40–44], some of which (e.g., CRF55_01B and CRF59_01B) have spread widely around China [45]. More recently, massive new recombinants composed of CRF01_AE and CRF07_BC (the two predominant strains among MSM populations) have also been reported around China [46–49]. However, most of them were unrelated in terms of phylogenetics, and a few were clustered but the origin and evolutionary relationship among them were not clear. We demonstrated, for the first time, that a group of closely related HIV-1 new recombinants in MSM may derive from one superinfection case, which supported the hypothesis of a model of generation of HIV-1 BF intersubtype recombinants with coincident breakpoints from South America [50].

Recently, early initiation of ART has become a worldwide public-health prevention strategy for HIV transmission. In China, the standards of ART initiation have been updated several times in national treatment guidelines [51–54]. In 2014, it was suggested that people with HIV infection with CD4+ T-cell count <500 cells/μL should receive ART. In 2016, it was suggested that all HIV-infected people, regardless of the CD4+ T-cell count, should be treated. Therefore, the putative donor reported in the present study did not start ART until 2014 according to the policy in China at that time. During the treatment-naïve period (~4 years), he became superinfected with another HIV strain and developed a series of CRF01_AE/CRF07_BC recombinants, then infected the other five recipients directly or indirectly.

Treatment-as-prevention approach has been shown to reduce the risk of HIV transmission in serodiscordant couples [55], and has been proposed and implemented in many countries (including China). Therefore, the prevalence of superinfection (such as in the donor in the present study) might be reduced. However, detection of increased URFs in areas with multiple HIV strains suggest there are many undiagnosed multiple-infected cases. Hence, if a multiple-infected case is diagnosed, not only should ART be started early to reduce the transmission risk, strengthened tracing of partners should also be done immediately. In this way, persons infected with complicated HIV-1 strains and a higher risk of further transmission can be diagnosed rapidly.

**Fig 5:**
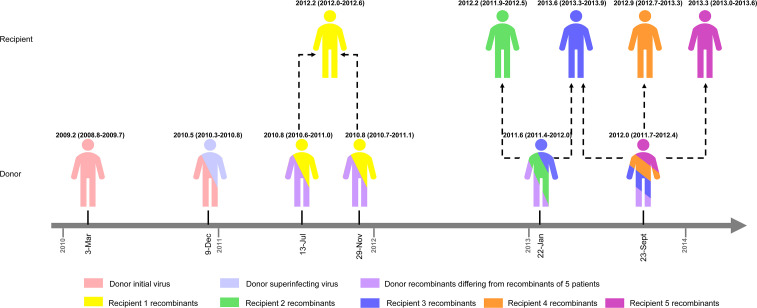
The presumed transmission relationship among six HIV-1-infected MSM. This hypothesis is based on the analysis of recombination forms and the estimated tMRCA of concatenated CRF01_AE and CRF07_BC segments of six HIV-1-infected patients. The superinfected patient is the putative donor and the other five patients are the putative recipients. The same color between donor and five recipients represents the similar or identical recombinants between them. The dotted line indicates that the recombinants might be transmitted directly or indirectly from the donor to putative five recipients. The tMRCA shown here is the mean tMRCA of concatenated CRF01_AE and CRF07_BC segments, 95% HPD is shown in parenthesis. tMRCA, time of the most common ancestor; 95% HPD, 95% highest probability density.

Due to protection of personal privacy, we did not have sufficient epidemiological data to determine the transmission chain among the six patients. However, we provided evidence from different perspectives to suggest there might be a direct or indirect transmission relationship among our six patients. We also inferred that the patient with HIV-1 superinfection might be the source of a lineage of closely related 0107 URFs (Fig 5).

Our study suggests an important role of HIV-1 superinfection on the generation and transmission of new recombinants. Furthermore, recombinants with high genetic similarity (but distinct recombination forms) could share a common origin. This observation provides a new perspective to infer the evolutionary relationship between HIV-infected individuals harboring recombinants. The present study also calls for greater attention to the monitoring, early ART, and strengthened management of multiple-infected individuals, including the tracing and testing of partners.

## Methods

### Ethics statement

This study was approved by the Ethics Committee of the First Affiliated Hospital of China Medical University ([2018] 2015-140-5). Written informed consent to participate in this study was obtained from all patients before sample collection.

### Study participants

The six study participants were newly diagnosed HIV-1-infected patients from a cohort of MSM in a voluntary HIV counseling and testing clinic of the First Affiliated Hospital of China Medical University. They were found to be infected with a lineage of CRF01_AE/CRF07_BC recombinant strains through phylogenetic analyses on *pol* sequences from routine genotypic testing for resistance to common anti-HIV drugs. The related laboratory testing has been described previously [56,57]. Donor (LNA819) was diagnosed with HIV-1 infection on 3 March 2010 and identified as having HIV-1 superinfection by next-generation sequencing [27]. Due to ART initiation or loss to follow-up, serial plasma samples between 2010 and 2013 were collected from the donor. One plasma sample at baseline was collected from recipient 3 (LN328575), recipient 4 (LN301538), and recipient 5 (LN328576), respectively. One plasma sample at the first or second HIV-positivity time-point after seroconversion was collected from recipient 1 (LN320639) and recipient 2 (LN320392), respectively.

### HIV-1 limiting-antigen avidity enzyme immunoassay (LAg-avidity EIA)

Plasma samples were tested for recent HIV infections with LAg-avidity EIA (Maxim Biomedicals, Rockville, MD, USA) according to manufacturer instructions. Normalized optical density (ODn) of 2.0 was used as a threshold cutoff to distinguish long-term HIV infection from recent HIV infection. Plasma with ODn upon initial screening >2 was classified as “long-term infection”, whereas that with ODn ≤2 was retested in triplicate for confirmation. Plasma with median ODn >1.5 was classified as “long-term HIV infection”, whereas that with ODn >0.4 but ≤1.5 was classified as “recent seroconversion”; for ODn ≤0.4, a serology confirmation test was necessary to further ensure that the plasma sample was HIV-positive.

### Amplification and sequencing of near-full-length genomes (NFLGs) and 3′ half-genomes

NFLGs and 3’ half-genomes were amplified and sequenced directly according to methods described previously [58]. In brief, HIV-1 RNA was extracted from 140-μL plasma sample using the QIAamp Viral RNA Mini Kit (Qiagen, Valencia, CA, USA) and was transcribed into complementary DNA using Superscript III Reverse Transcriptase (Life Technologies, Carlsbad, CA) with primer 07Rev8 (5′-CCTARTGGGATGTGTACTTCTGAA CTT-3′, nt 5193–5219) and 1.R3.B3R (5′-ACTACTTGAAGCACTCAAGGCAAGCTTTATTG-3′, nt 9611–9642) for 5′ half-genome and 3′ half-genome, respectively. The sequences of the 5′ half-genome and 3′ half-genome were amplified by nested polymerase chain reaction (PCR) by single-genome amplification (SGA) using 1 unit of Platinum Taq DNA Polymerase High Fidelity (Life Technologies, Carlsbad, CA) according to manufacturer instructions. The primers used to amplify the 5’ half-genome in the first round of PCR was 172 (5′-ATCTCTAGCAGTGGCGCCCGAACAG-3′, nt 625–649) and 07Rev8, and in the second round of PCR was 174 (5′-CTCTCGACGCAGGACTCGGCTTGCT-3′, nt 683–707) and Rev11 (5′-ATCATCACCTGCCATCTGTTTTCCAT-3′, nt 5041–5066). The primers used to amplify the 3’ half-genome in the first round of PCR was 07For7 (5′-CAAATTAYAAAA ATTCAAAATTTTCGGGTTTATTACAG-3′, nt 4875–4912) and 2.R3.B6R (5′-TGA AGCACTCAAGGCAAGCTTTATTGAGGC-3′, nt 9607–9636), and in the second round of PCR was VIF1 (5′-GGGTTTATTACAGGGACAGCAGAG-3′, nt 4900–4923) and Low2C (5′-TGAGGCTTAAGCAGTGGGTTCC-3′, nt 9591–9612). The thermal cycling conditions for PCR were one cycle at 94°C for 2 min, followed by 35 cycles of 94°C for 15 s, 60°C for 30 s, 68°C for 5 min, followed by one cycle of extension at 68°C for 10 min. Positive amplification products were purified and sequenced directly using internal walking primers by BGI (Beijing, China).

### Phylogenetic and recombination analyses

Sequences were assembled with Sequencher 5.4.6 (Gene Codes, Ann Arbor, MI, USA), and aligned using Gene Cutter within HIV databases (www.hiv.lanl.gov), then adjusted manually with BioEdit 7.0 (www.mbio.ncsu.edu/ BioEdit) [59]. All sequences obtained in this study were blasted in the local sequence library by our research team. We used BLAST within HIV databases (www.hiv.lanl.gov) to eliminate potential cross-contamination during the experiment. Reference sequences were downloaded from the Los Alamos HIV Database (www.hiv.lanl.gov). Maximum likelihood phylogenetic trees (ML trees) of the aligned NFLG, 3′ half-genomes, and concatenated CRF01_AE and CRF07_BC segments were constructed by Fast Tree [60] and edited by Fig Tree v1.4.2 (http://tree.bio.ed.ac.uk/software/figtree). Recombination analyses were first done with the Recombinant Identification Program (RIP) [61] and jumping profile Hidden Markov Model (jpHMM) within HIV databases (www.hiv.lanl.gov) [62]. Further confirmation was achieved with bootscanning in Simplot 3.5.1 [63] to define the recombination structures (window size: 350 nt; step size: 50 nt; bootstrap replicate: 250).

### Bayesian Markov Chain Monte Carlo (MCMC) evolutionary analyses

We wished to explore the phylogenetic relationship and the time of the most recent common ancestor (tMRCA) of viruses from the six participants, Bayesian phylogenetic analyses were done using the MCMC inference implemented in BEAST v2.5.1 [64]. For concatenated CRF01_AE segments, strict molecular-clock analyses were undertaken under the model of general time reversible (GTR) + I+G nucleotide substitution. For concatenated CRF07_BC segments, relaxed molecular-clock analyses were undertaken under the model of Tamura Nei 93 (TN93) nucleotide substitution. The MCMC chains were run 200-million times and sampled every 20,000 steps. The output was tested for convergence using Tracer v1.6, and related parameters were estimated from an Effective Sample Size (ESS) more than 200. Phylogenetic trees were summarized using TreeAnnotator (with 10% burn-in) and then edited using Fig Tree v1.4.2.

### GenBank accession numbers

The NFLG and 3’ half-genome sequences reported here are available in GenBank under accession numbers KX434794, KX434795, KX434797, KX434798 KX434799, MT857722, MW287665-MW287747, and MW344769-MW344807.

## Supporting information

S1 Fig Highlighter plots of HIV-1 3′ half-genome diversity in six HIV-1-infected MSM. The initial strain (CRF01_AE) and superinfected strain (CRF07_BC) from the donor were chosen as master sequences and are colored light-coral and slate-blue, respectively. The x-axis represents the base number. The y-axis represents the sampling dates of donor or recipients. The 3’ half-genome sequences obtained from the donor and five recipients are shown in panel A and B, respectively. Some recombination key sites were marked with ↑.
(TIF)

S2 Fig Distribution of breakpoints across HIV-1 3’ half-genome sequences of all recombinant viruses (N = 88) from the donor and five recipients. The initial strain (CRF01_AE) and superinfected strain (CRF07_BC) were chosen as parental strains. SimPlot v3.5 (window size = 300 nt; step size = 10 nt) was used to identify breakpoint locations. In general, 13 breakpoints were identified on the HIV-1 3’ half-genome and labeled by a to m, respectively. The x-axis represents the range of breakpoints. The y-axis represents the frequency of all recombinant viruses at the corresponding breakpoints. Distribution of 13 breakpoints on the HIV-1 3’ half-genome (HXB2: 4950–9589) (schematic) are below.
(TIF)

S3 Fig Maximum likelihood (ML) trees of HIV-1 CRF01_AE/CRF07_BC recombinants from the donor and five recipients. The same sequences as in the Bayesian Evolutionary Analysis Sampling Trees (BEAST) analysis (Fig 4) were chosen to construct ML trees. ML trees for concatenated CRF01_AE segments (regions V+VII + IX) (A) and CRF07_BC segments (regions IV+VI) (B) were constructed under the model of substitution of GTR+I+G sites.
(TIF)
