# Supplementary figures and images for: First complete-genome documentation of HIV-1 intersubtype superinfection with transmissions of diverse recombinants over time to five recipients
Source: PLoS Pathog. 2021 Feb 12;17(2):e1009258. doi: 10.1371/journal.ppat.1009258 (PMC7906459; doi:10.1371/journal.ppat.1009258)

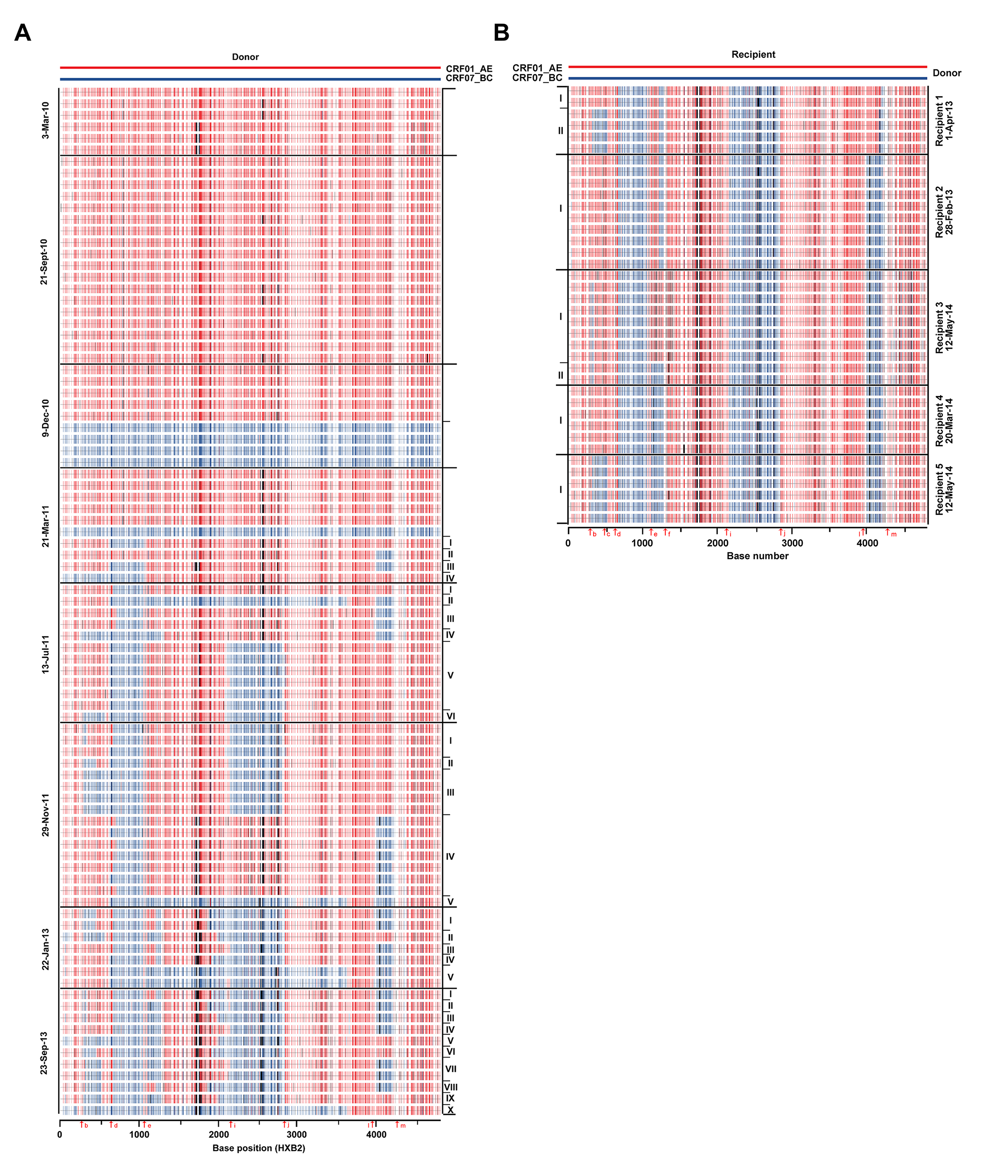

Supplement: S1 Fig — The initial strain (CRF01_AE) and superinfected strain (CRF07_BC) from the donor were chosen as master sequences and are colored light-coral and slate-blue, respectively. The x-axis represents the base number. The y-axis represents the sampling dates of donor or recipients. The 3’ half-genome sequences obtained from the donor and five recipients are shown in panel A and B, respectively. Some recombination key sites were marked with ↑. (TIF) [file ppat.1009258.s001.tif]

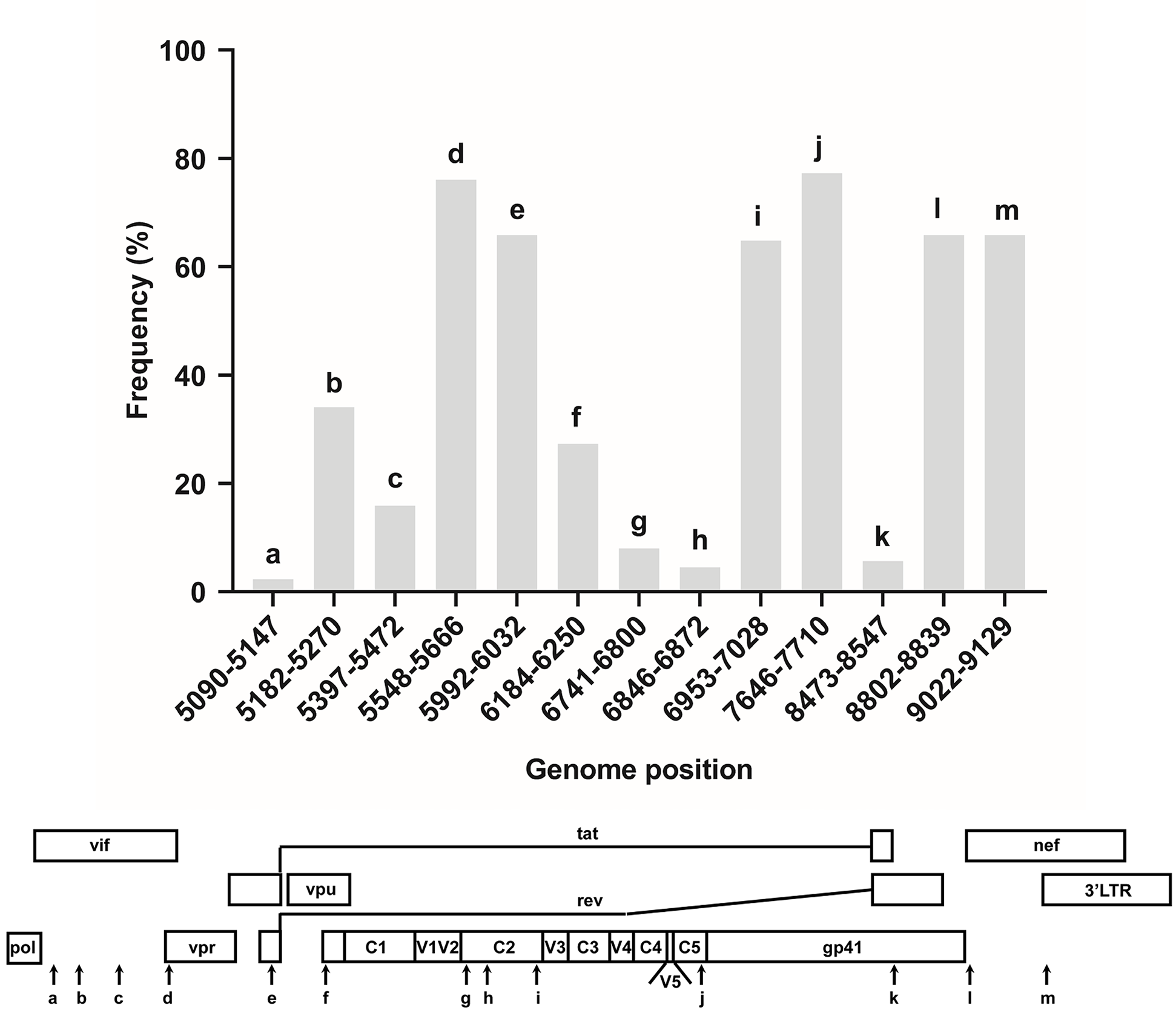

Supplement: S2 Fig — The initial strain (CRF01_AE) and superinfected strain (CRF07_BC) were chosen as parental strains. SimPlot v3.5 (window size = 300 nt; step size = 10 nt) was used to identify breakpoint locations. In general, 13 breakpoints were identified on the HIV-1 3’ half-genome and labeled by a to m, respectively. The x-axis represents the range of breakpoints. The y-axis represents the frequency of all recombinant viruses at the corresponding breakpoints. Distribution of 13 breakpoints on the HIV-1 3’ half-genome (HXB2: 4950–9589) (schematic) are below. (TIF) [file ppat.1009258.s002.tif]

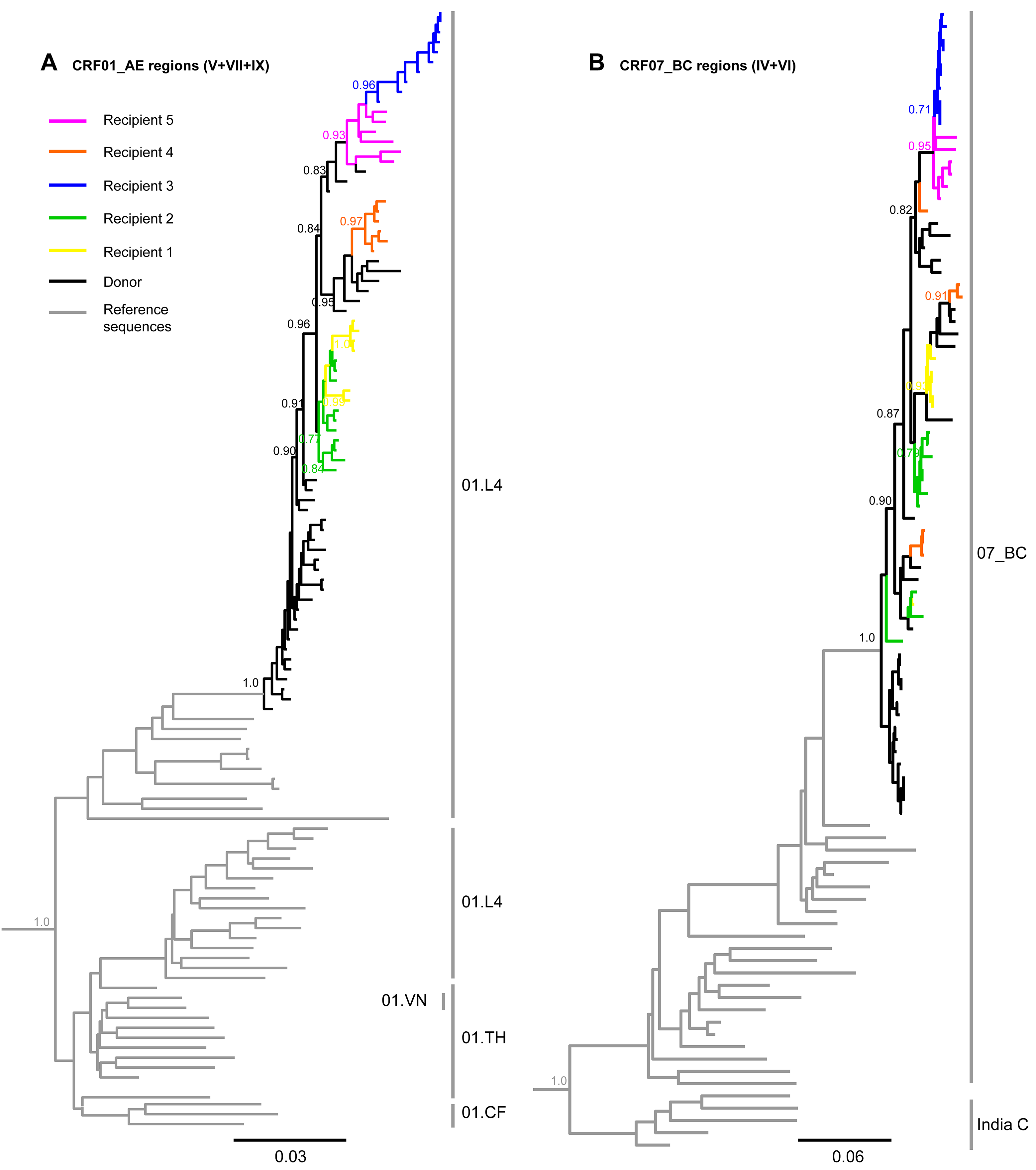

Supplement: S3 Fig — The same sequences as in the Bayesian Evolutionary Analysis Sampling Trees (BEAST) analysis (Fig 4) were chosen to construct ML trees. ML trees for concatenated CRF01_AE segments (regions V+VII + IX) (A) and CRF07_BC segments (regions IV+VI) (B) were constructed under the model of substitution of GTR+I+G sites. (TIF) [file ppat.1009258.s003.tif]
